# 

**Published:** 1874-02

**Authors:** 


### Contents of February Number.

### ORIGINAL DEPARTMENT.

ART. I.—Old Eyes made New. By Charles A. Robertson, M. D.. ...... 241

ART. II.—Medical Society of the County of Albany. Semi-Monthly Meeting, Jan. 14th and Jan. 28th, 1874. Reported by F. C. Curtis, M. D., Secretary,........ 245

ART. III.—Concentrated and Palatable Powders. By J. W. Southworth, M. D....... 264

### MISCELLANEOUS.

Buffalo State Insane Asylum. Remarks of Dr. James P. White,........ 267

The Nitrite of Amyl in Epilepsy,........ 269

On Prof. Esmarch's Mode of performing Bloodless Operations, ......... 270

On the Importance and Dangers of Rest in Pulmonary Consumption,.... 272

### EDITORIAL DEPARTMENT.

Twenty-Eighth Annual Commencement of the Buffalo Medical College,.. 273

Buffalo City Dispensary,........ 274

Defective Drainage,........ 275

State Medical Society,........ 275

New Journal,........ 275

Books Reviewed:

A Hand Book of the Theory and Practice of Medicine. By F. T. Roberts, M. B., B. Sc.,........ 276

On the Cerebral Convolutions of Man. By Alexander Ecker, 276

A Treatise on Diseases of the Eye. By J. Soelberg Wells, F. R. C. S.,........ 277

Lacerations of the Female Perineum and Vesico-Vaginal Fistula. By D. Hayes Agnew, M. D.,........ 277

Sex in Education. By Edward H. Clarke, M. D.,.......... 278

The Mechanism of the Ossicles of the Ear. By H. Helmholtz,........ 279

Books and Pamphlets Received........ 280

